# Two-Year-Olds’ Symbolic Use of Images Provided by a Tablet: A Transfer Study

**DOI:** 10.3389/fpsyg.2019.02891

**Published:** 2019-12-20

**Authors:** Daniela Jauck, Olga Peralta

**Affiliations:** ^1^Instituto Rosario de Investigaciones en Ciencias de la Educación, Consejo Nacional de Investigaciones Científicas y Técnicas de Argentina (CONICET), Rosario, Argentina; ^2^Facultad de Psicología y Relaciones Humanas, Universidad Abierta Interamericana (UAI), Rosario, Argentina

**Keywords:** symbolic use, images, tablet, means of communication, sources of information, 2-year-old children

## Abstract

Touch screen devices are nowadays part of everyday life and parents and educators assume that young children use their images in a symbolic way. This research aims at replicating and expanding a previous one that reported that, contrary to expectations and to studies with printed or video images, 2-year-old children used the images on a touch screen tablet to retrieve a hidden object (Search), but not to communicate the location of an object they have observed being hidden (Point). In this research, we carried out a transfer study comparing the performance of an Experimental (Search-Point) and a Control group (Point-Point). First, we found that the Experimental group outperformed the Control group. Second, we found that the successful symbolic previous experience gained in the Search tasks was transferred to the Point task, task which 2-year-olds otherwise fail.

Understanding the symbolic nature of images and learning to use them is an important cognitive challenge, not only in childhood but throughout life (Ittelson, 1996). Images can be found everywhere in our environment in printed as well as in touch screen devices. Touch screen devices are increasingly present in daily life, and adults as well as children use them in a variety of ways, for communicating, playing, searching for information, and so forth. Due to the attractiveness and ease of manipulation, adults take for granted that children understand and use the images in a tablet in a symbolic way (Rideout and Hamel, 2006; Vittrup et al., 2016).

However, in order to use an image symbolically, children have to form two mental representations simultaneously, one of the image as an object and the other one as the entity the image represents (DeLoache, 1987). This dual representation hypothesis has been tested in numerous studies using search tasks. In the original task (DeLoache, 1987), the experimenter gave the child information concerning the symbolic relationship that connected a small-scale model with the full-size room that the model represented. Then, the experimenter hid a miniature toy in a particular location in the scale model. Next, without the child observing, she hid a similar toy in the full-size room and then asked the child to find it. The results showed that 2½-year-old children did not use the scale model as a source of information to find the toy, that is, as a representation of the actual room. However, 6 months later, at 3 years of age, most children solved the task.

An extensive body of research established that it is easier for children to take images as symbols than three-dimensional objects (DeLoache, 1991; Marzolf and DeLoache, 1994). Three-dimensionality affects dual representation. When young children are in front of a three-dimensional symbolic object, like a scale model or a replica, they tend to see it only as an object and not as a representation (DeLoache, 2000). Even though the images are also objects, they are much less salient as such than three-dimensional ones.

In sum, research has shown that 3-year-old children succeed when the information concerning the hiding location is provided by a scale model. However, when the same information is provided by a printed or video image, children succeed at 2½ years of age, but not at 2 years of age (DeLoache and Burns, 1994; Troseth and DeLoache, 1998; Schmitt and Anderson, 2002; Peralta and Salsa, 2009).

Using a somehow reverse procedure than the one of the search task, Peralta and Salsa (2009) reported that 2-year-olds pointed on a photograph the location of an object they have observed being hidden. After this successful experience, the authors (Peralta and Salsa, 2009) also reported that 2-year-olds used the photograph as a source of information to find a hidden object, task which 2-year-olds without this previous experience fail.

Previous experience has been proposed as a mechanism for promoting a general expectation or readiness to look for symbolic relations among entities (DeLoache, 2002). Evidence supporting this hypothesis comes from studies that tested transfer from an easier task to a more difficult version of it. These studies have been carried out within symbolic media, e.g., from more iconic scale models to less iconic models (DeLoache et al., 2004), as well as across symbolic media, e.g., from pictures to scale models (Marzolf and DeLoache, 1994).

The images displayed on tablets are non-traditional symbolic means because they are interactive (Sheehan and Uttal, 2016). Tablets differ from traditional desktop computers because they are, like toys, light and mobile. Their use stimulates visual, auditory, tactile, and kinesthetic sensory systems providing instantaneous feedback. In this sense, research has proposed that even though the physical contingency of touch screens may promote symbolic understanding (Kirkorian and Pempek, 2013), differential effects depend on children’s age (Choi and Kirkorian, 2016; Kirkorian et al., 2016). The interactive features of the touch screen may distract the children from the task, inviting children to use the tablet as a three-dimensional attractive toy rather than focusing on the representations on the screen (Sheehan and Uttal, 2016).

Research has reported that 2-year-old children used the images displayed on a tablet as a source of information to find a hidden object, but they did not use the images as a means of communication to inform the location of a hidden object. These findings are opposite to what was expected and reported on printed photographs (Peralta and Salsa, 2009). The authors argued that, on the one hand, the images on a tablet may stress the reality-image correspondence and highlight the intention of representation on the part of the user. However, on the other hand, the three-dimensional characteristics of the device itself, plus the fact that the screen responded with a little movement and sound when children pointed, could have underscored the device as an object, obscuring the symbolic function of its images to communicate information.

The purpose of the research presented here was to further investigate the use of images displayed on a tablet in two symbolic tasks by 2-year-old children. For that purpose, we replicated and expanded the findings reported by Jauck and Peralta (2016) and investigated transfer effects of previous experience from one task to the other. Transfer effects were tested between two different symbolic tasks.

In one task, children have to use the images in a tablet as a source of information to find a hidden object on a real space (Search task). In the other task, children have to communicate the location of an object they have observed being hidden in the space (Point task). The Search task consisted of an adaptation of DeLoache’s (1987) object retrieval task. The Point task was an adaptation of the task used by Peralta and Salsa (2009) in which the children had to point in a photograph the location of an object they have observed being hidden in a real room.

We hypothesize that the findings reported by Jauck and Peralta (2016) will be replicated in the sense that 2-year-olds will succeed using the image on the tablet in the Search task but not in the Point task. Also, a successful previous experience using the images on a tablet as a source of information is expected to be transferred when used as a means of communication, task which 2-year-olds fail.

## Method

### Participants

Forty 2-year-old children were randomly assigned to two groups: (1) Experimental group: 20 children (Mean age = 25.00 months; SD *=* 1.17; range = 23–27), 8 girls and 12 boys and (2) Control group: 20 children (Mean age = 24.95; SD *=* 1.05; range = 23–27), 10 girls and 10 boys. The children were contacted through the daycare center they attended. Informed consent from parents and institutions was obtained. All parents had completed high school; most parents (54 out of 80; 32 mothers and 35 fathers) had university studies and worked in their professions or in commercial activities. Three (out of 40) mothers did not work outside their home. The research was carried out according to ethical standards.

### Materials

A portable room (1 m high × 80 cm depth × 1 m width) served as the space where a doll, named Lily, was hidden. The portable room contained several hiding locations: a bed, a box, an armchair, a bedside table, and cushions. We also used a tablet (10.1″) that produced a little flash of light and sound when the screen was touched, as most touch screens usually do. Supplementary Figure S1 shows a front view of the hiding room and the tablet with the image of the room on its screen.

### Procedure

We adapted two tasks. The Search task was an adaptation of the classic object retrieval task (DeLoache, 1987; DeLoache and Burns, 1994), in which the experimenter hid a toy in the room without the child observing and then indicated to the child the hiding location on the image of the tablet. The child had to find the object in the room using the information provided by the tablet. The Point task was inspired by a task previously used with printed photographs (Peralta and Salsa, 2009). In this task, the experimenter hid the object in the room while the child observed, then asked the child to point on the tablet screen image the location where the object was hidden. Each task comprised four trials in which the toy was hidden in four different locations. The order of presentation of the locations was counterbalanced. Children completed two consecutive blocks of four trials each.

Transfer effects were investigated by comparing the performance of an Experimental group and a Control group. The Experimental group completed four Search tasks and immediately afterward four Point trials; the Control group completed eight Point trials. This Control was introduced in order to check if possible changes in performance were due to learning or familiarization effects with the materials/procedures and/or to the interference of one task with the other.

The tasks consisted of two phases:

1. Orientation: Its purpose was to familiarize the children with the materials and the activities to be carried out in the test. First, the experimenter showed the child the doll that was going to be hidden. She then placed the doll in all hiding locations of the room: bed, box, bedside table, cushions, and armchair, as she named them. Afterward, she showed the tablet to the child and took one picture of the whole room and of two of different pieces of furniture. Then, she placed the tablet with the image on its screen next to each one of the pieces of furniture marking the correspondence; e.g., “Look at this photo of Lily’s table (showing the image on the tablet), and this is Lily’s table (indicating the piece of furniture)”. Finally, the experimenter placed the doll on the bed, took a picture and said: “This picture shows you where Lily is; remember that it will tell you where Lily is!” The purpose of this step was to highlight the intention with which the images were going to be used in the task.
2. Task
  - Search: The experimenter told the child, “I’ll hide Lily somewhere in her house; but you don’t have to look while I do so! Then I’ll show you in the photo where she is hiding and you will go look for Lily.” The child turned around not looking at the hiding event. Once Lily was hidden, the experimenter together with the child took a picture of the entire room saying: “Lily is hiding here (indicating the location on the image but not naming it), go find her in her house!”
  - Point: The experimenter said: “I’ll hide Lily in her house, look!” while hiding the toy somewhere in the room, the child observed the hiding action. Then, the experimenter said: “Let’s take a picture of Lily’s house so you can show me where she is hiding” and she took a picture of the room. Afterward, they both turned around (with the room out of their view) and, showing the tablet to the child, the experimenter asked: “In this picture, can you point where I hid Lily?” Immediately after that, the experimenter asked the child to look for Lily, “Now, let’s go find Lily in her house!” and the child had to find the toy in the room. This last step controlled for memory, as the child might have failed to point to the correct location simply because she/he had forgotten where the toy had been hidden.

### Strategy of Analysis

The dependent variable was the number of correct responses; percentages are also reported to facilitate comparisons. A response was considered correct if the child pointed to the hiding location (Point task) or found the hidden object (Search task) on the first attempt. It was also considered correct if the child pointed/searched in the previous location but immediately self-corrected, without the experimenter’s intervention. The response was considered incorrect if the child pointed/searched in the wrong location or if he/she did not point or search at all. Neither no Search nor no Point responses were observed. Perseverative responses were also recorded since it has been repeatedly reported that the most common error in this type of tasks is to select the immediate previous location (O’Sullivan et al., 2001; Sharon and DeLoache, 2003; Suddendorf, 2003). In addition, the correct searches in the memory segment of the Point task were also considered. Preliminary analyses showed no gender effects.

We also examined individual performance based on the criterion set for successful participants, which was solving at least three of the four trials of each block.

Due to the size of the sample and since no normality was assumed, we opted for a non-parametric analysis using the *Mann-Whitney U* test for independent samples (between groups) and the *Z-Wilcoxon* test for related samples (within groups).

## Results

In line with the results reported by Jauck and Peralta (2016), we found that children showed a much better performance searching than pointing: Block 1 Experimental group–Search (*n* = 51; 64% correct trials) vs. Block 1 Control group–Point (*n* = 11; 14% correct trials) (*U* = 50.50; *p* < 0.0001) (see Supplementary Figure S2).

Transfer effects were tested with two different comparisons. First, the performance of the Experimental group on Block 2 (Point task) was compared with the performance of the Control group on Block 1 (Point task) to verify that prior experience with the Search task was critical to succeed in the Point task. Results showed that when children first solved the Search task, their performance in the Point task improved. Significant differences were found in pointing with or without a previous experience in searching: Block 2 Experimental group (*n* = 37; 46% correct trials) vs. Block 1 Control group (*n* = 11; 14% correct trials) (*U* = 105.00; *p* < 0.007).

Next, we compared the performance of the two groups in Block 2 (Point task) to examine whether the transfer effect was over and above the effect of experience with materials and procedures. The performance of the Experimental group (*n* = 46%) was significantly higher than chance (*X*^2^ = 34.45, gl 1, *p* < 0.0001) and significantly better than the one of the Control group (*n* = 15; 15%) (*U* = 109; *p* < 0.009). Even though the performance of the Control group (Point-Point) slightly improved from Block 1 (14%) to Block 2 (15%), no significant differences were found (*Z* = 0.250, *ns*); therefore, this improvement cannot be considered the consequence of transfer effects.

We also analyzed *individual performance*. In the Control group, one child met the criterion for a successful participant in Block 1 (Point) and three in Block 2 (Point). In the Experimental group, 12 children met the criterion in Block 1 (Search), and nine in Block 2 (Point); of these nine children, eight were successful in Block 1, demonstrating transfer at the individual level.

Regarding *perseverative responses*, the children virtually never pointed to or searched in the immediate previous location. Two perseverative responses in pointing and two in searching were observed. However, as children immediately self-corrected, without any intervention from the experimenter, all were coded as correct.

Concerning the *memory test*, in the Point task segment of the Experimental group, children retrieved the toy from the hiding location in 84 trials (93%). As for the Control group, children retrieved the toy in 71 trials (89%) of the first block, and in 77 trials (96%) of the second. Once more, these data show that children’s failure in the Point task cannot be attributed to having forgotten where the toy had been hidden, but to failure in representing the real room through the displayed image on the tablet.

## Discussion

The purpose of this research was to investigate the symbolic use of images on a tablet by 2-year-old children. We used two tasks in which the images function either as a means of communication (Point task) or as a source of information (Search task). We found that children used the images as a source of information but not as a means of communication. We also found that a prior successful experience in the Search task significantly improved performance in the Point task, illustrating a transfer effect.

The properties of the device used and the specific characteristics of the tasks may be one possible explanation for these results. The image on a tablet is two-dimensional, but the device itself is three-dimensional. As it has been demonstrated, three-dimensionality affects dual representation (DeLoache, 1987, 2000). Also, three-dimensional symbolic objects are more likely to be treated as objects of action rather than of contemplation and reflection (Striano et al., 2001; Gelman et al., 2008).

Furthermore, in the Point task, children touched the screen obtaining a movement and sound response, some children even explored the edges of the tablet before pointing. In this sense, it has been shown that manipulation affects symbolic access (Uttal et al., 2009; Tare et al., 2010). In the Search task, children never touched the screen, they just observed the image to obtain information for finding the hidden object.

Children’s successful performance in the search task replicates the results obtained in a previous study (Jauck and Peralta, 2016). However, the results of the current study contrast with most studies with printed or video images that found 2-year-olds fail to connect images with referents in this task (DeLoache, 1987; DeLoache and Burns, 1994; Troseth and DeLoache, 1998; Schmitt and Anderson, 2002; Peralta and Salsa, 2009).

Instantly capturing the images with the tablet may not only have helped children establish correspondences between real objects and images but also may have enabled children to get the intention of using the images in the task. As shown, children improve their performance when they capture the intention of a symbolic tool in a particular task (Salsa and Peralta, 2007; Chen and Siegler, 2013).

It is also worth noting that children made no perseverative errors. These errors have been reported in almost all search task studies with young children (e.g., O’Sullivan et al., 2001; Peralta and Salsa, 2003, 2009; Sharon and DeLoache, 2003). One possible explanation could lie in that embedding a picture on a tablet before each new search favors updating the information, reducing perseverative errors (Suddendorf, 2003).

The findings of the present research contrast with the results reported in a previous study (Peralta and Salsa, 2009) in which similar tasks were assigned to the children but using printed photographs. In that study, the photographs were used as a means of communication but not as a source of information.

The discrepancy of the results may partly rest in the medium used. Children are very familiar with printed pictures; joint picturebook reading is a very common activity in their lives. In these interactions, adults communicate information about objects, people, or events (Ninio and Bruner, 1978; Peralta, 1995; Fletcher and Reese, 2005). Thus, the familiarity of children with the communicative function of images on paper possibly facilitates their symbolic use as a means of communication. Nowadays, images provided by touch screen devices are part of young children’s lives, even though their function as a means of communication is not nearly as common as the one of photographs or drawings. Children usually use touch screen devices to do recreational activities on their own (Barkin et al., 2006), which probably lead them to consider tablets as toys, not paying attention to the representational nature of their images.

We also found that while children initially did not use images on the tablet to communicate a real observed situation (Point task), prior successful experience in the use of images as a source of information (Search task) significantly improved performance. In line with DeLoache (2002), we propose that children transferred a symbolic awareness, conceived as general expectation or readiness to look for and detect symbolic relations among entities. The effect of symbolic experience has been demonstrated in studies where children managed to solve more difficult tasks after solving highly analogous simpler ones (Marzolf and DeLoache, 1994; DeLoache et al., 2004). In the current research, we illustrated transfer effects, but between two different symbolic tasks.

A limitation of the present research concerns the use of touch screen devices, not when the adult manipulates the device, but when children do so to “discover” and take advantage of their interactive properties. In this sense, contingent or non-contingent experiences using touch screens have been described as having differential effects in children’s symbolic understanding and use (Troseth et al., 2016; Troseth and Strouse, 2017). These differential effects were also found to be dependent on age (Choi and Kirkorian, 2016; Kirkorian et al., 2016).

Finally, as Sheehan and Uttal (2016) noted, research on dual representation suggests that the manipulative features of touch screens might make it difficult for young children to use them as a symbolic medium; however, their unique physical interactive affordances may help overcome this difficulty. Future studies can address this important and key question testing the impact of not only physical interactivity with the device but also social interactivity with more experienced partners.

## Data Availability Statement

All datasets generated for this study are included in the article/Supplementary Material.

## Ethics Statement

The studies involving human participants are the ones reviewed and approved by Consejo Nacional de Investigaciones Científicas y Técnicas of Argentina. Written informed consent to participate in this study was provided by the participants’ legal guardian/ next of kin.

## Author Contributions

This research is part of the DJ’s doctoral thesis under the supervision of the OP. DJ carried out all the data collection. Both DJ and OP designed the studies, analyzed the results, and wrote the article.

### Conflict of Interest

The authors declare that the research was conducted in the absence of any commercial or financial relationships that could be construed as a potential conflict of interest.

## Supplementary Material

SUPPLEMENTARY FIGURE S1 Photograph of the hiding room and the tablet.

SUPPLEMENTARY FIGURE S2 Children’s correct responses by block and group.
