# Supplementary figures and images for: Two-Year-Olds’ Symbolic Use of Images Provided by a Tablet: A Transfer Study
Source: Front Psychol. 2019 Dec 20;10:2891. doi: 10.3389/fpsyg.2019.02891 (PMC6932993; doi:10.3389/fpsyg.2019.02891)

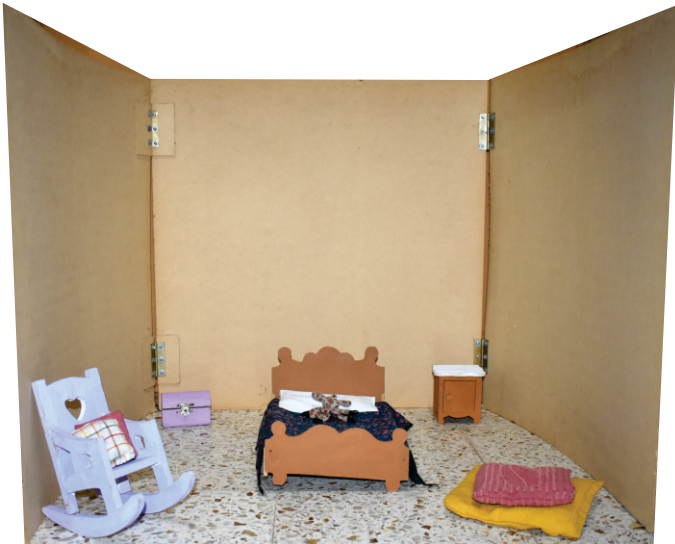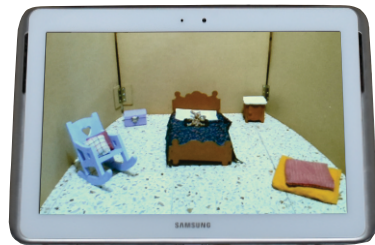

Supplement: SUPPLEMENTARY FIGURE S1 — Photograph of the hiding room and the tablet. [file Data_Sheet_1.PDF]

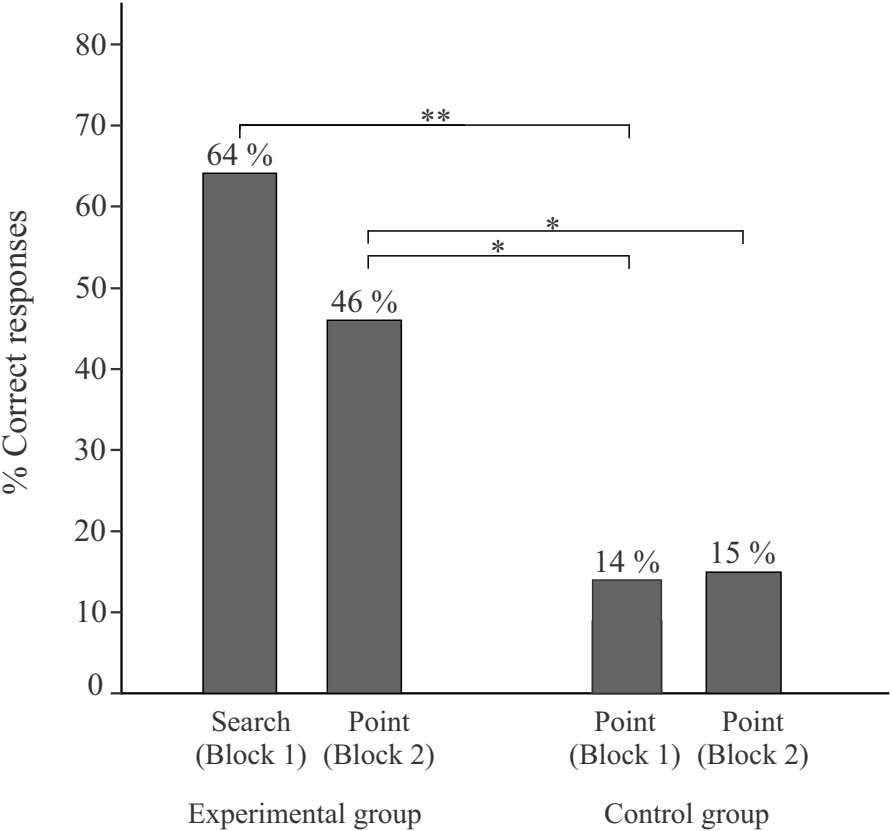

\* $p < 0.01$

\*\* $p < 0.0001$

Supplement: SUPPLEMENTARY FIGURE S2 — Children’s correct responses by block and group. [file Data_Sheet_2.PDF]
